# Application of the theory of planned behaviour for predicting the determinants of workplace violence reporting behaviour among public hospital healthcare workers in Malaysia: A cross-sectional study

**DOI:** 10.51866/oa.312

**Published:** 2023-10-29

**Authors:** Halimatus Sakdiah Minhat, Mohammad Nafis Sahiran

**Affiliations:** 1 MBBCh BAO, MPH, DrPH, Malaysian Research Institute on Ageing, Universiti Putra Malaysia, Serdang, Selangor, Malaysia.; 2 Department of Community Health, Faculty of Medicine and Health Sciences, UPM, Serdang, Selangor, Malaysia. Email: halimatus@upm.edu.my; 3 MPH, DrPH State Health Office, Ministry of Malaysia, Melaka, Malaysia.

**Keywords:** Workplace violence, Abuse reporting, Healthcare workers

## Abstract

**Introduction:**

Reporting workplace violence (WPV) is a crucial preventive measure. Given the great impact of WPV on mental health and well-being, this study aimed to determine the prevalence and determinants of WPV reporting among healthcare workers (HCWs).

**Method:**

A total of 557 public hospital HCWs in Melaka were recruited via probability sampling. A questionnaire guided by the theory of planned behaviour was developed, pre-tested and distributed. Malaysians with a minimum employment period of 12 months who experienced WPV within the same period in the selected public hospitals were eligible for inclusion. Multiple logistic regression analysis was conducted to determine the association between the independent variables and WPV reporting.

**Results:**

Psychological violence was the most common WPV (80.3%), with only 177 (31.8%) respondents reporting such. The respondents who had high subjective norm (adjusted odds ratio [AOR]=2.160, 95% confidence interval [CI]=1.32–3.53) and perceived behavioural control scores (AOR=3.976, 95% CI=2.41–6.55); were clinical (AOR=2.679, 95% CI=1.43–5.02) and non-clinical (AOR=4.271, 95% CI=2.23–8.18) support staff; experienced physical WPV (AOR=13.157, 95% CI=3.83–45.24) and both physical and psychological WPV (AOR=2.029, 95% CI= 1.13–3.65); and perceived that WPV was intentional (AOR=11.111, 95% CI=6.50–19.00) were more likely to report WPV.

**Conclusion:**

HCWs who experience physical WPV have the highest likelihood to report, followed by those who perceive WPV as intentional. The prevalence of reported WPV among public hospital HCWs is low, potentially underestimating its true occurrence owing to underreporting. Ensuring readily available reporting mechanisms for WPV, especially the psychological type, is crucial for HCWs.

## Introduction

Workplace violence (WPV) refers to any incidents related to staff being assaulted, abused or threatened in work-related circumstances, including commuting to and from work, either directly or indirectly challenging their safety, well-being or health. It is commonly categorised into physical or psychological violence, which can manifest as verbal abuse, harassment, bullying and threat^1^ and even escalate to homicide.

The magnitude of WPV experienced by healthcare workers (HCWs) varies by country, setting, study design and questionnaire. A related review on 136 articles critically evaluating the prevalence of violence among nurses revealed non-physical violence being the commonest type of WPV (70%), followed by bullying, physical violence and sexual harassment.^2^ A higher prevalence of WPV has been reported in Southeast Asian regions: 54.1%,^3^ 54.6%^4^ and >70%^5^ in Thailand, Indonesia and Singapore, respectively.

WPV negatively affects not only the victims but also the healthcare system. Victims of WPV have been reported to experience an increased prevalence of work-related stress symptoms and depression and reduced job satisfaction and commitment, directly compromising the quality of healthcare provided.^1,6^ WPV is postulated to have more detrimental effects in developing countries,^7^ such as Malaysia, owing to the lack of information and awareness on WPV contributed by the perceived or social norms of underreporting WPV.^8^ Reporting WPV is crucial, but underreporting is common, leading to numerous undesirable impacts particularly on the psychological wellbeing of workers.

Underreporting WPV is prevalent in the literature.^9-13^ Its prevalence reached as high as 88%-93.5% in some studies.^14,15^ This phenomenon is also seen worldwide in healthcare professions.^15-17^ Survey-based assessments showed that 21%-50%^16^ and 26.6% of victims^17^ submitted formal reports regarding violence. A much lower prevalence of reporting WPV was documented in another research: Only 10.9% of victims of physical WPV and 6.5% of victims of psychological WPV submitted formal reports.^15^ Underreporting impedes violence preventive measures and efforts by underrating the true magnitude of the problem.^18^ It also leads to the impression that less prevention is needed than may be warranted,^18^ implicating the potential limitation towards preventive efforts.^19,20^

Underreporting is associated with numerous causes and factors, but which are mainly related to behaviour, leading to its complexity. Some international studies have shed some light on the issue. The lack of injury or time lost^21,22^ caused by WPV as well as the tiring and timeconsuming incident reporting procedures^21^ are among the commonest reasons behind underreporting of WPV. Further, the lack of superior or co-worker support, fear of retaliation or accusation,^21,22^ uncertainty of the positive outcome of reporting^15^ and common perception that violence is simply part of the job^21,23^ also contribute towards the increasing prevalence of underreporting among HCWs. The lack of agreement on the definitions of violence within organisations and employees^14,22^ may also influence reporting behaviour. For instance, the absence of physical injury may not be perceived as violence.^21^ HCWs may not report incidents if they perceive that patients do not mean any harm - the aggressive incident is related to their illnesses.^14,22^ Sociodemographic factors such as age, sex, ethnicity, educational level, occupation and years of employment are also implicated as factors associated with reporting behaviour, although conflicting results are sometimes reported.^14,17,24,25^ These factors associated with underreporting of WPV have not been thoroughly examined in the local setting of Malaysia.

WPV among HCWs has received limited coverage among scholars in Malaysia. A recent survey on 231 HCWs based at the emergency department of public hospitals in Melaka revealed a considerably high 6-month prevalence of WPV (38%).^26^ A different study among female nurses in the same setting reported a prevalence of sexual harassment of 51.2% and an incidence in the past year of 22.8%,^27^ indicating the increasing severity of this issue among nurses. Retrievable published research regarding this issue in Malaysia is currently limited to quantitative surveys on the prevalence of WPV in general rather than on reporting or underreporting of incidents, creating a gap in the understanding and development of effective strategies designed to reduce underreporting of WPV.

Reporting WPV is a complex phenomenon influenced by personal misperceptions. Various theories have been used extensively in understanding diverse events and behaviours. This study employed the theory of planned behaviour (TPB),^28^ a framework widely used to predict human behaviours. According to the theory, the intention to perform a certain behaviour and subsequently the actual behaviour are influenced by attitudes, subjective norms and perceived behavioural control (PBC). The applicability of the TPB has been investigated to understand whistleblowing behaviour or reporting of malpractice within organisations. The results have demonstrated a strong correspondence between its constructs in predicting such behaviour.^29^ The theory has also been successfully utilised in previous research to predict the intention to report crimes.^30^ A study among hospital nurses in Israel showed significant correlations between intention to report and behavioural attitudes, subjective norms, self-efficacy and reporting behaviours.^17^

The present study aimed to identify the prevalence of WPV reporting among HCWs and the predictors of WPV reporting behaviour by applying the TPB. The TPB has been used widely in numerous research not only to prognosticate intentions to exhibit particular health-related behaviours^31,32^ but also to understand participation in ethical behaviours rather than unethical behaviours.

## Methods

This cross-sectional study was conducted from October 2020 to March 2021 among HCWs from all three public hospitals in the state of Melaka. HCWs included staff working with patients and their relatives or with blood or other bodily fluids from patients in their working environment: doctors, clinical support staff such as nurses and medical assistants and non-clinical support staff such as ambulance drivers, health attendants, science officers, medical laboratory technicians, pharmacists, radiographers, physiotherapists and administrative or clerical workers.

The required sample size was calculated using the two-independent proportions formula,^33^ adjusting for comparison between two groups and anticipating a 10% nonresponse rate. The estimated sample size was 611 HCWs, who were then recruited via proportionate stratified random sampling in all three hospitals. However, only 557 eligible respondents completed the distributed questionnaire. A random number table was used to sample the HCWs until the required number of samples needed for each stratum was achieved. Only Malaysian citizens who were working in the hospitals under study for at least 12 months and experienced WPV within the past 12 months were included in this study. The dependent variable was the reported WPV. The independent variables included the TPB constructs (i.e. behavioural intention, behavioural attitude, subjective norm and PBC related to the reported WPV), sociodemographic profile (i.e. age, sex, race/ethnicity and educational level), work profile (i.e. occupation and years of experience in the healthcare sector) and WPV-related data (i.e. type of encountered WPV, identity of the perpetrator and perceived intention of the perpetrator).

The self-administered questionnaire was pretested, validated in Malay language and divided into three sections: personal and workplace (Section A), WPV and reporting (Section B) and TPB data (Section C). All sections were developed by the researchers. In particular, Section C was developed using a guideline for constructing TPB-based questionnaires.^34^ The respondents’ sociodemographic and work profiles were recorded in Section A.

Section B extracted information about the respondents’ WPV experience and reporting behaviour, including the type of WPV (i.e. physical violence, psychological violence or both), identity of the perpetrator (i.e. patient/client, relatives of patient/client, staff member, management, supervisor, general public or others), perceived intention of the perpetrator (i.e. intentional or unintentional) and formal reporting (i.e. yes or no) as well as the reason for non-reporting.

The TPB constructs were assessed in Section C. Each question was scored using a 7-point Likert scale. Behavioural intention was evaluated with four items, attitude with 10 items, subjective norm with nine items and PBC with 10 items. Given that the data were not normally distributed, median scores were calculated as the cut-off point to divide low and high behavioural intention, low (negative) and high (positive) attitude, low and high subjective norm and low and high PBC scores.

The content validity of the questionnaire was evaluated by a panel of experts familiar with WPV. Face validity testing was conducted among 65 HCWs from a neighbouring hospital. The questionnaire was distributed in Malay language, with the translation process performed per the World Health Organization guideline. The internal consistency of the questionnaire was measured using Cronbach’s alpha coefficients. The Cronbach’s alpha coefficient of the final questionnaire for behavioural attitude, subjective norm and PBC was 0.816, 0.866 and 0.691, respectively. The Cronbach’s alpha coefficient for behavioural intention was 0.510, which was above the 0.5 acceptable level. Generally, it is difficult to obtain a higher value if the construct has fewer than 10 items.^35^ Data were analysed using IBM SPSS version 26. Multiple logistic regression was used to identify the predictors of WPV.

Ethical approval was obtained from the national institutional review board. Permission from all hospital directors was obtained prior to the commencement of this study. Each respondent completed a written consent form, with an information sheet containing information about the purpose of the study. Confidentiality was maintained at all stages of the study.

## Results

Table 1 shows the background characteristics of the respondents and their distributions according to the TPB constructs. The prevalence of reported WPV in the last 12 months was 31.8%, with the physical type being the commonest. Psychological violence was the most common unreported WPV. The majority of the respondents were Malay (86.9%), were women (66.2%), were aged 30–39 years (44.9%), were diploma holders (43.6%), worked in clinical support groups (37.9%) and had a working experience of less than 5 years (68.1%). The scores for each TPB construct were almost equally distributed among the respondents.

**Table 1:** Background characteristics of the respondents (N=557).

Variable	Frequency (%)	Median (IQR)
*Age (year)*		33 (9)
≤29	184 (33.0)	
30-39	250 (44.9)	
40-49	102 (18.3)	
≥50	21 (3.8)	
*Sex*		
Male	188 (33.8)	
Female	369 (66.2)	
Race/ethnicity		
Malay	444 (79.7)	
Chinese	55 (9.9)	
Indian	45 (8.1)	
Others	13 (2.3)	
Educational level		
Primary	2 (0.4)	
Form 3	6 (1.1)	
Form 5	87 (15.6)	
Form 6	15 (2.7)	
Diploma	243 (43.6)	
Bachelor	182 (32.7)	
Master and above	22 (3.9)	
*Occupation*		
Doctor	149 (26.8)	
Clinical support staff	211 (37.9)	
Non-clinical support staff	197 (35.4)	
*Working experience (year)*		8 (10)
<5	191 (34.3)	
5-9	145 (26.0)	
10-19	172 (30.9)	
≥20	49 (8.8)	
*Behavioural intention towards WPV reporting*		4.67 (1.33)
Low (<4.67)	309 (55.5)	
High (≥4.67)	248 (44.5)	
*Behavioural attitude towards WPV reporting*		99.25 (52.63)
Low (<99.25)	276 (49.6)	
High (≥99.25)	281 (50.4)	
*Subjective norm towards WPV reporting*		41.71 (44.32)
Low (<41.71)	280 (50.3)	
High (≥41.71)	277 (49.7)	
*PBC towards WPV reporting*		6.25 (21.50)
Low (<6.25)	275 (49.4)	
High (≥6.25)	282 (50.6)	
*WPV reporting*		6.25 (21.50)
Yes	177 (31.8)	
Physical	20 (76.9)	
Psychological	113 (25.3)	
Both	44 (57.6)	
No	380 (68.2)	
Physical	6 (23.1)	
Psychological	334 (74.7)	
Both	44 (52.4)	

IQR= Inter quartile range; PBC= Perceived behavioural control; WPV= Workplace violence.

Table 2 shows the distribution of the respondents according to the violence type, perpetrator’s identity and perceived perpetrator’s intention. Almost all WPV experienced by the respondents was in the form of psychological violence (80.3%), perpetrated by a patient/client (44.7%) and perceived as intentionally perpetrated (52.6%).

**Table 2:** Distribution of the violence type, perpetrator’s identity and perceived perpetrator’s intention (N=557).

Variable	Frequency (%)
*Violence type*	
Physical	26 (4.7)
Psychological	447 (80.3)
Both	84 (15.1)
*Perpetrator's identity*	
Patient/client	249 (44.7)
Relative of patient/client	102 (18.3)
Staff member	94 (16.9)
Management	9 (1.6)
Supervisor	33 (5.9)
Public	12 (2.2)
0thers	0 (0.0)
Combination^*^	58 (10.4)
*Perceived perpetrator's intention*	
Intentional	293 (52.6)
Unintentional	264 (47.4)

* Includes more than one perpetrator at the same time

The results of the multiple logistic regression analysis are shown in Table 3. Only subjective norms and PBC significantly predicted WPV reporting behaviour. The respondents with high subjective norm and PBC scores were 2.2 (adjusted odds ratio [A0R]=2.160, 95% confidence interval [CI] = 1.32-3.53, P=0.002) and 4.0 times (AOR=3.976, 95% CI=2.41-6.55, P<0.001) more likely to report WPV, respectively, than those with low scores. Additionally, the clinical and non-clinical support staff were 2.7 (AOR=2.679, 95% CI=1.43-5.02, P=0.002) and 4.3 times (AOR=4.271, 95% CI=2.23-8.18, P<0.001) more likely to report WPV, respectively, than the doctors. The respondents who experienced physical WPV and both physical and psychological WPV and those who perceived WPV to be intentional were 13 (AOR=13.157, 95% CI=3.83-45.24, P<0.001), 2.0 (AOR=2.029, 95% CI=1.13-3.65, P=0.018) and 11 times (AOR=11.111, 95% CI=6.50-19.00, P<0.001) more likely to report WPV, respectively, than their counterparts.

**Table 3:** Predictors of WPV reporting among public hospital healthcare workers in Melaka.

Multiple logistic regression					
Variable	B	AOR	P	95% CI	
				Lower	Upper
Intercept	-4.673	0.009	<0.001		
*Subjective norm towards reporting of WPV*					
Low		1.0			
High	0.770	2.160	0.002^*^	1.32	3.53
*Perceived behavioural control towards reporting of WPV*					
Low		1.0			
High	1.380	3.976	<0.001^*^	2.41	6.55
*Occupation*					
Doctor		1.0			
Clinical support staff	0.986	2.679	0.002^*^	1.43	5.02
Non-clinical support staff	1.452	4.271	<0.001^*^	2.23	8.18
*WPV type*	
Psychological		1.0			
Physical	2.577	13.157	<0.001^*^	3.83	45.24
Both	0.708	2.029	0.018^*^	1.13	3.65
*Perceived perpetrator's intention*					
Unintentional		1.0			
Intentional	2.408	11.111	<0.001^*^	6.50	19.00

* Significant at P<0.05

B = regression coefficient Forward logistic regression was applied. Multicollinearity and interaction terms were checked. Hosmer and Lemeshow test: P=0.922, classification table: overall percentage=81.0%, Cox and Snell R squared=0.340, Nagelkerke R squared=0.476, R0C=0.869

## Discussion

### Prevalence of reported WPV

This study empirically confirmed that underreporting is a significant problem among HCWs, as evidenced by the low prevalence of reported WPV. This prevalence observed during the 12-month study period is comparable with other reports: Approximately 21%–50%^16^ and 26.6%^17^ of victims submitted formal reports regarding WPV. In contrast, the prevalence of reported WPV in this study is higher than that in a previous related study among nurses: Only 10.9% of victims of physical WPV and 6.5% of victims of psychological WPV submitted formal reports to their leaders or other designated officers.^15^ This variation could be attributed to the differing working sites and occupations of the respondents in each study, which may influence their perception and judgement towards WPV as well as the decision to report the incident. Patient-on-worker violence may be considered as common in dealing with job challenges or even part of the job^36^ among HCWs but not among administrative staff or lecturers. Workers’ interpretation and evaluation of the severity or intensity of the violent incident might preclude reporting. The lack of documentation due to underreporting has been a major challenge in combating WPV in healthcare settings.

While the low prevalence of reported WPV in this study was derived from hospital settings, it is comparable with the prevalence of reported WPV in studies conducted at primary care settings: 44.3%^37^ and 46.7%.^38^ This comparison provides insights into the similarity of the prevalence of reported WPV across different healthcare settings, essential in understanding the scope of WPV.

### Predictors of WPV

In this study, only subjective norms and PBC were found to significantly predict WPV reporting behaviour among the respondents. The clinical and non-clinical support staff, respondents who experienced physical WPV or both physical and psychological WPV and respondents who perceived WPV to be intentional were also more likely to report WPV. Various factors influenced the HCWs’ decisions to report or ignore instances of WPV. These factors can be related to individual workers’ experiences (e.g. fear of retaliation, lack of physical injury and inconvenience), work (e.g. the misperception that exposure to violence is part of the job and that fear affects customer satisfaction scores) and organisation (e.g. managers’ attitude).^36^

This study confirmed the role of subjective norms towards reporting of WPV reported in another TPB-based related research. A significant positive correlation between subjective norms and intention to report WPV was noted in this previous study among nurses, with a high level of intention to report subsequently predicting a higher actual prevalence of reporting WPV.^17^ Individual behaviours can be influenced by what is perceived as a norm in a certain community or group of people.^29^ When a group is committed to making WPV reporting decision, group members are also likely to take the same action and vice versa. Hence, eliminating false perceptions or subjective norms regarding WPV can create an environment that enables victims to report WPV.

PBC was another factor found to influence WPV reporting behaviour among the HCWs in this study. This finding is supported by another report that PBC was significantly positively correlated with the intention to report WPV and that a high level of intention to report subsequently predicted a higher actual prevalence of reporting WPV than did a low level of intention to report. 17 In a study exploring the effects of perceived control on the outcomes of workplace aggression and violence through a series of moderated regression analyses, perceived control was directly associated with emotional well-being and indirectly associated with somatic health and neglect, suggesting the enhancement of perceptions of control through training targeting WPV.^39^

In terms of occupation, the clinical and non-clinical support staff showed a higher likelihood of reporting WPV than did the doctors. The finding aligns with another report that doctors were less likely to report physical WPV than nurses.^25^ Doctors often viewed themselves as holding top hierarchical status and worthy of respect from patients, colleagues and the general public.^40^ Consequently, they were more likely to feel ashamed of reporting violent incidents, considering them trivial and less important than patients’ deteriorating health status.^41^

The decision to report WPV was also predicted by the type of violence. The HCWs who experienced physical violence were more likely to report WPV than those who experienced psychological violence, similar to previous findings.^24^ This is likely due to the prevalence of specific types of violence among HCWs, to the extent that they may mistakenly perceive it as normal and consequently fail to report all forms of violence.^42^ HCWs tend to ignore less serious psychological violence (e.g. light verbal abuse) and eventually accept it as part of their job.^17^ This perception should be intervened early to discourage even more rampant extreme WPV from happening as stipulated in the broken window theory, wherein insensitivity and ignorance towards minor crimes create a conducive territory towards a more serious crime. Furthermore, although psychological violence is the predominant type, there is still controversy in distinguishing it from other types,^43^ creating a grey area that can affect reporting decisions.

Lastly, this study revealed that perceiving the violence as intentional predicted reporting behaviours. This is because victims tend to appraise the perpetrators’ profile to judge whether they can be held responsible. The level of intention to report perpetrators with a psychiatric diagnosis or those who appeared confused was lower than its counterparts, as they might be perceived as not committing the violence intentionally.^17^ Patients’ diagnosis and presenting conditions or comorbidities increased HCWs’ empathy towards patients and their family and friends.44 For patients or their visitors, the respondents cited a wide range of factors that could be considered excusable.^16^ This collective perception among HCWs should be corrected to help them understand that reporting of unintentional WPV does not equate to punishing the perpetrator but rather to improving the factors contributing to such incidents.

The abovementioned findings may hold implications beyond hospital settings, particularly in primary care centres. Understanding the nuanced reasons behind underreporting can help in formulating tailored strategies that foster a safer environment for HCWs in diverse settings. Primary care settings, which often involve different dynamics between patients and HCWs compared with hospital settings, may pose unique challenges in addressing and reporting WPV. Further research should be conducted in primary care settings to explore and ascertain this issue, identify challenges and develop tailored strategies to combat such. These efforts are crucial to ensure the well-being of HCWs and the quality of care across various healthcare domains, from those working in primary care settings to those working in tertiary healthcare centres. In essence, while these findings offer valuable insights, their direct applicability to primary care settings may require further investigation and consideration of the unique factors at play in such environments.

### Strengths and limitations

Among the strengths of this study is the involvement of a wide range of occupational categories in the public hospitals while achieving a high response rate. This provides a variety of views from the respondents, thus permitting a more holistic understanding of WPV reporting among the population. Another strength is that the use of a locally developed, validated and reliable questionnaire ensured that the collected data were of high quality and appropriate for the local study population. The use of the TPB as the guiding framework for the research demonstrates a scholarly approach to translating theory into practice. Additionally, probability sampling enables the findings to be generalised to a broader HCW population with a similar background.

This study also presents certain limitations. The employment of a cross-sectional study design precluded establishing a causal or temporal relationship between the independent and dependent variables, necessitating caution when drawing conclusions. The research also relied solely on the recollection and honesty of the respondents regarding their reporting behaviour for the past 12 months, potentially introducing recall bias. Furthermore, the use of a self-administered questionnaire is another potential source of bias, as the respondents might have conformed to social desirability. In a previous related study comparing selfreport and actual documentation of hospital incidents, 23% of respondents stated that they self-reported violent incidents, but only 4% of them had done so.^14^ Despite these acknowledged limitations, the insights gained from this research into the extent of underreporting may still prove valuable in arousing interest in this topic.

### Recommendation for future studies

A qualitative or mixed-method study design is recommended to further explore and identify other factors or explain the results for a deeper understanding of the issue. Objective measurements of reporting behaviour, such as direct first-hand observations or triangulation with official documentations, are also suggested. These approaches could improve the accuracy of the results and reduce response bias due to social desirability or recall bias. Further, research with a larger sample size and involvement of other healthcare professions in different settings, such as HCWs in health clinics, district health offices and private sectors, could provide valuable views regarding WPV reporting behaviour among these populations. Another recommendation for future studies is to use a study design that can identify a cause-and-effect association (e.g. cohort design). Future researchers are also suggested to explore other theories or models to provide supplementary information pertaining to WPV to ensure full coverage of areas that might be limitedly investigated by the TPB. The mediating effect of the intention constructs of the TPB should also be considered in future studies to further explain the application of the theory in understanding WPV reporting behaviour.

### Prevention and policy implications

This research highlights multiple key findings that can be used to lay out several short-and long-term plans to reduce underreporting of WPV among HCWs. In the short term, stakeholders should implement intervention programmes for doctors such as continuous medical education (CME) and WPV simulation. These programmes should be conducted multiple times at intervals deemed as appropriate, feasible and sustainable. It is also recommended for junior or freshly graduated doctors to be briefed on the overall WPV issue among HCWs, the importance of reporting WPV and the WPV reporting process.

The study also highlights the importance of subjective norms and PBC towards WPV reporting. Individual perception can be influenced by sociodemographic background and beliefs prevalent in one’s environment. Accordingly, it is imperative to incorporate health education initiatives, including CME, courses and training about WPV. These efforts should occur more frequently to enhance HCWs’ resources, skills and capabilities while rectifying any misconceptions that WPV is an inherent part of their job. WPV-related training must be made compulsory alongside other relevant orientation modules and materials for newly employed HCWs. Interventions based on theories, particularly the TPB, should be developed through collaboration with subject matter experts, including human psychology and behaviour experts, WPV experts, academicians and frontliners. The aim is to change the perception of HCWs regarding WPV while boosting their confidence in overcoming barriers, thereby influencing not only their own behaviour but also those in their immediate circle.

Psychological WPV should receive focused attention, as it comprises the largest proportion of WPV experienced by HCWs and is also the most underreported. HCWs must be explicitly informed that they are not only permitted but also expected to report any instances of psychological WPV they encounter to facilitate proper investigation and intervention.

In the long term, the normalisation of reporting WPV should be the objective of interventions, with a particular focus on higher-ranking HCWs, such as doctors and supervisors. Focusing on these HCWs is crucial because healthcare professions often follow an apprentice-type system, wherein the attitudes and behaviours of senior staff have a substantial influence in shaping the culture.

Strengthening WPV policies can further promote the normalisation of WPV reporting by incorporating policy implementation into the key performance indices of stakeholders. The commitment and support of higher-ranking stakeholders are crucial in cultivating and nurturing the right perceptions regarding WPV among HCWs, ultimately enhancing their WPV reporting behaviour. Additionally, the establishment of a neutral third party to investigate WPV reports can alleviate concerns about being reprimanded, blamed, alienated or labelled as whistleblowers for those choosing to report violent incidents. This approach also provides victimised higher-ranking HCWs a channel to relay their personal WPV experiences, which can improve the reporting behaviour among this group.

## Conclusion

This study found that psychological violence was the commonest type of WPV among the HCWs, and the prevalence of WPV reported was considerably low. The HCWs who had high subjective norm and PBC scores, were working as clinical and non-clinical support staff, experienced physical WPV and both physical and psychological WPV and perceived WPV as intentional were more likely to report the incident. Health education and intervention aiming to foster better awareness towards reporting WPV should target HCWs at risk of underreporting, particularly those experiencing psychological WPV. Additionally, qualitative studies to explore the experiences of individual victims are needed, as WPV can be a sensitive issue for some to disclose.

## Data sharing statement

Only available upon request.

How does this paper make a difference in general practice?

- Workplace violence (WPV) is an ongoing highly preventable public health issue.
- The study provides usefUl information related to reporting and underreporting of WPV among healthcare workers (HCWs).
- The findings provide theoretical evidence on the potential factors associated with WPV among HCWs.
- The findings may serve as a basis for future research and planning for preventive interventions to reduce WPV among HCWs.
